# Comparison of the frequency of bacterial and viral infections among children with community-acquired pneumonia hospitalized across distinct severity categories: a prospective cross-sectional study

**DOI:** 10.1186/s12887-016-0645-3

**Published:** 2016-07-22

**Authors:** Amanda C. Nascimento-Carvalho, Olli Ruuskanen, Cristiana M. Nascimento-Carvalho

**Affiliations:** Bahiana School of Medicine, Bahiana Foundation for Science Development, Salvador, Bahia Brazil; Department of Paediatrics, Turku University and University Hospital, Turku, Finland; Department of Paediatrics, Federal University of Bahia School of Medicine, Salvador, Bahia Brazil

**Keywords:** Acute respiratory infection, Lower respiratory tract infection, Lung disease, Respiratory viruses, Severity assessment

## Abstract

**Background:**

The comparison of the frequencies of bacterial and viral infections among children with community-acquired pneumonia (CAP) admitted in distinct severity categories, in an original study, is lacking in literature to-date. We aimed to achieve this goal.

**Methods:**

Children aged 2-59-months-old hospitalized with CAP were included in this prospective study in Salvador, Brazil. Clinical data and biological samples were collected to investigate 11 viruses and 8 bacteria. Severity was assessed by using the World Health Organization criteria.

**Results:**

One hundred eighty-one patients were classified as “non-severe” (*n* = 53; 29.3 %), “severe” (*n* = 111; 61.3 %), or “very severe” (*n* = 17; 9.4 %) CAP. Overall, aetiology was detected among 156 (86.2 %) cases; viral (*n* = 84; 46.4 %), bacterial (*n* = 26; 14.4 %) and viral-bacterial (*n* = 46; 25.4 %) infections were identified. Viral infection frequency was similar in severe/very severe and non-severe cases (46.1 % *vs*. 47.2 %; *p* = 0.9). Pneumococcal infection increased across “non-severe” (13.2 %), “severe” (23.4 %), and “very severe” (35.3 %) cases (qui-squared test for trend *p* = 0.04). Among patients with detected aetiology, after excluding cases with co-infection, the frequency of sole bacterial infection was different (*p* = 0.04) among the categories; non-severe (12.5 %), severe (29.3 %) or very severe (55.6 %). Among these patients, sole bacterial infection was independently associated with severity (OR = 4.4 [95 % CI:1.1–17.6]; *p* = 0.04) in a model controlled for age (OR = 0.7 [95 % CI:0.5–1.1]; *p* = 0.1).

**Conclusions:**

A substantial proportion of cases in distinct severity subgroups had respiratory viral infections, which did not differ between severity categories. Bacterial infection, particularly pneumococcal infection, was more likely among severe/very severe cases.

**Electronic supplementary material:**

The online version of this article (doi:10.1186/s12887-016-0645-3) contains supplementary material, which is available to authorized users.

## Background

Community-acquired pneumonia (CAP) continues to be a major cause of death among children aged under-5 years worldwide and almost all of these deaths occur in developing countries [1]. In the 1980s, it was established that most children dying from CAP in these regions had bacterial infection. Such assumption was based on the results of bacterial culture of lung aspirates from cases in these developing countries, as well as of lung aspiration studies and post-mortem studies from cases in developed countries in the pre-antimicrobial era [2, 3]. During the last decade, a wide range of studies have been conducted across different continents with the purpose of investigating the aetiology of childhood CAP [4]. Of note, while in the former set of studies only conventional bacteriological methods were employed, new molecular tests have been used in recent investigations, which consequently increased the yield of microbiological results [5, 6]. In this latter group, children with different categories of severity have been included, either with non-severe, severe, or very severe disease [4]. However, the comparison of the frequencies of different aetiologies among children with CAP in distinct severity categories in an original study is lacking in literature to-date.

In this context, we assessed whether there was difference in the frequency of bacterial and viral infections among children with CAP in distinct severity subgroups.

## Methods

This was a prospective cross-sectional study conducted at the Federal University of Bahia Hospital, in Salvador, North-eastern Brazil, where community-dwelling children from the low income population were first seen at the hospital’s Emergency Room. This is a public service. Data were prospectively collected from September 2003 to May 2005. CAP diagnosis was based on the report of respiratory complaints and fever or difficulty breathing plus the detection of pulmonary infiltrate or pleural effusion on the chest radiograph taken at the initial evaluation and read by the paediatrician on duty. For the purpose of this analysis, only children with tachypnoea or chest indrawing were included. Every child under-5-years-old hospitalized due to CAP diagnosis at the Emergency Room, based on the aforementioned criteria, was enrolled. Those with chronic lung disease except asthma, underlying comorbidities (cancer, organ dysfunction like cardiac, hepatic, respiratory or renal insufficiency), any other concurrent infections (for example, measles, chickenpox, tuberculosis, whooping cough, otitis, gastroenteritis, pyodermitis, meningitis), suspected or diagnosed immunodeficiency (including children born to an HIV-infected mother), or transferred from other health-care units were excluded. The applied exclusion criteria assured that only previously healthy children were enrolled and the clinical characteristics were exclusively attributable to the current CAP episode.

Upon hospital admission, demographic and clinical data and biological samples were collected by the research team, after receiving written informed consent by parents or legal guardians. Detailed interviews regarding the current illness, general medical history and thorough physical examinations were performed to complete a standardized questionnaire and assure eligibility. Data on clinical evolution and outcome were not collected because treatment may influence them. Therefore, the focus of this investigation was the severity presented by patients upon admission to hospital. Nasopharyngeal aspirates (NPA) collected through the nostrils were stored (−70 °C) until virologic tests were performed. Blood was collected for blood culture (BACTEC automatic system), buffy-coat (-70 °C) and serum (−20 °C) storage. Follow-up visits occurred between 2 and 4 weeks after enrolment when blood was also collected for serum storage (−20 °C). This procedure allowed the comparison of specific IgG titres as an investigational tool to detect several aetiologies. Everyone in the study had to have both NPA and paired serum samples collected to assure the same opportunity for investigation of all pathogens included in the panel.

Nineteen aetiological agents were investigated: 11 viruses and 8 bacteria. *Streptococcus pneumoniae*, *Haemophilus influenzae*, and *Moraxella catarrhalis* infections were investigated by blood culture and by searching for specific IgG increase tested by an in-house enzyme immune assay (ELISA) in paired serum samples. For pneumococcal infections, IgG antibodies to pneumococcal pneumolysin and pneumococcal C-polysaccharide were used; a ≥2-fold or ≥3-fold increase, respectively, in antibody titres, between paired serum samples, was considered diagnostic [7].

For *H. influenzae* and *M. catarrhalis* infections, Ig (polyvalent) antibodies against whole bacterial cell antigens were measured and a ≥3-fold antibody increase between paired serum samples was considered diagnostic [7]. Pneumococcal infection was also sought by pneumolysin-polymerase chain reaction (PCR) performed in acute buffy-coat for the detection of pneumococcal DNA [8]. *Staphylococcus aureus* infection was investigated by blood culture. *Mycoplasma pneumoniae* infection was investigated by testing for specific IgM by using a commercial ELISA kit (Platelia, Bio-Rad, Marnes La Coquette, France) [9]. *Chlamydia trachomatis* IgG antibodies were measured by a commercial, solid-phase ELISA (Ani Labsystems Ltd., Vantaa, Finland). The laboratory diagnosis was based on signal to cut-off (S/CO) values, which were ≥1.4 S/CO [10]. An in-house microimmunofluorescence (MIF) test was used to measure IgG, IgA and IgM antibodies to *Chlamydophila pneumoniae* and *Simkania negevensis*, using purified, formalized elementary bodies of strains Kajaani 6 in *C. pneumoniae* and ATCC strain Z (ATCC, Catalog no. VR-1471) in *S. negevensis* tests. The diagnosis was based on a ≥4-fold increase in IgG or IgA antibodies between the paired serum samples or on the presence of IgM antibodies (a titre of ≥10) [11]. Rhinovirus, enterovirus, and human metapneumovirus were investigated by reverse transcriptase PCR in NPA. Parainfluenza virus 1, 2, 3, respiratory syncytial virus (RSV), influenza virus A and B, and adenovirus were investigated by viral antigen identification in the NPA by time-resolved fluoroimmunoassay with monoclonal antibodies; additionally, virus-specific serum antibody titres were determined in paired serum samples using an ELISA with an antigen-coated solid phase and horseradish peroxidase conjugated rabbit anti-human IgG; a ≥3-fold antibody titres increase was considered diagnostic [12]. Human bocavirus was investigated by quantitative PCR of NPA and serum, IgG increase determination in paired serum samples and searching for IgM and IgG avidity by ELISA [13]. Frequencies of these aetiological agents analysed by age distribution have been published [14–17].

The patients were grouped into three distinct severity subgroups according to the World Health Organization (WHO) severity criteria for children aged 2 months and above available in the 2000 decade; patients with “non-severe” CAP had tachypnoea neither with chest indrawing nor with any danger signs, patients with “severe” CAP had chest indrawing or supraclavicular recession without any danger signs, and patients with “very severe” CAP presented any danger signs, which were inability to drink, convulsions, lethargy, stridor in a calm child, or central cyanosis [18]. Tachypnoea was defined as respiratory rate ≥50 breaths/min in children aged 2–11 months and respiratory rate ≥40 breaths/min in children aged 12–59 months [18]. Therefore, children aged under-2 months, or without tachypnoea, or with missing respiratory rate, or missing danger signs were excluded. Severity classification was performed by a researcher blinded to the aetiological tests’ results, who had previously received training regarding the WHO severity criteria. Additionally, the patients were further grouped into two distinct severity subgroups according to the current WHO severity criteria published in 2013 [19]. According to these latter criteria, children with ¨non-severe¨ pneumonia have tachypnoea or chest wall indrawing, and children with ¨severe¨ pneumonia have central cyanosis, stridor in a calm child, inability to drink, lethargy, or convulsions, besides having tachypnoea or chest wall indrawing. That is, the subgroups “non-severe” and “severe” in the former criteria were grouped into the subgroup “non-severe” in the latter criteria; the subgroup labelled “very severe” in the former criteria was re-named “severe” in the latter criteria. Invasive disease was defined as positive blood culture (bacteraemia) or positive blood pneumococcal PCR.

For the purpose of analysis, the cases were classified as viral infection when only viral infection was detected, bacterial infection when bacterial infection was diagnosed irrespective of also viral infection having been detected; this last subgroup was split into two others: typical bacterial infection if infection by *S. pneumoniae*, *H. influenzae*, *M. catarrhalis* or *S. aureus* was found irrespective of other agents; atypical bacterial infection if infection by *M. pneumoniae*, *C. trachomatis*, *C. pneumoniae* or *S. negevensis* was detected irrespective of viral infection.

Continuous variables showed non-parametric distribution and were presented as median [interquartile range (IQR)]. Categorical variables were compared using chi-square or Fisher’s exact test, as appropriate. A trend analysis for bacterial, typical bacterial, and pneumococcal infection across 3 different severity categories was performed using qui-squared test for trend. Continuous variables were assessed using Mann–Whitney U. Analysis was stratified by age. Multivariable logistic regression analysis by enter method assessed the potential impact of bacterial infection (predictor variable) on clinical severity upon admission (outcome variable) in an age adjusted model. Statistical tests were two-tailed, at significance level of 0.05. SPSS software (version 9.0, IBM, Armonk, New York) was used for analysis. Sample size was estimated considering a smaller frequency of bacterial infection in non-severe cases of 30 % and an expected frequency of bacterial infection in severe/very severe cases of 45 %. Thus, the sample size was estimated as 162 cases, considering a significance level of 0.05 (95 % Confidence Interval [95 % CI]) and power of 80 %. Exclusion criteria were chosen to address potential confounders. Blinding at severity classification was performed to address potential bias. Cases with any missing biological sample or data were excluded.

Different data from the same research project have already been published. The novelty in this paper is the presentation of clinical characteristics which were used to group the cases in distinct severity categories, along with the analysis of the aetiological data across these distinct severity categories.

## Results

**Fig. 1:**
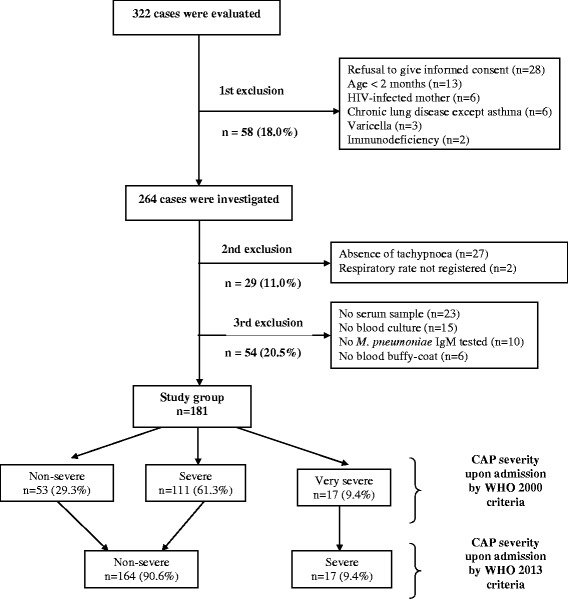
Flow-chart of recruitment of children hospitalized with community-acquired pneumonia referred for aetiology investigation and grouped according to severity upon admission

Overall, 322 patients were evaluated, out of which 141 (43.8 %) were excluded due to different reasons (Fig. 1). Therefore, the study group comprised 181 patients (Fig. 1). There were no additional diagnoses as reasons for hospitalization. The overall median (IQR) age was 17 (10–27) months and there were 113 (62.4 %) boys. No patient had previously received either pneumococcal or influenza vaccines. On the contrary, 85.0 % had received *H. influenza*e type b vaccine according to information retrieved from their vaccination cards. Cough (98.9 %), fever (96.7 %), and difficulty breathing (86.1 %) were the most frequent complaints. Antibiotic use in the previous 72 h (21.3 %) and asthma (23.9 %) were reported.

The patients were classified as non-severe (*n* = 53; 29.3 %), severe (*n* = 111; 61.3 %), or very severe (*n* = 17; 9.4 %) CAP (Fig. 1) in accordance with the WHO severity criteria available in the 2000 decade. Table 1 shows demographic and clinical characteristics of patients in each severity subgroup. Children with non-severe CAP were older than those with severe or severe plus very severe CAP. No significant differences were found among distinct severity subgroups in regard to duration of illness prior to hospitalization. Likewise, no difference was found when the frequency of asthma was compared between children with non-severe or severe plus very severe CAP (23.1 % *vs*. 24.2 %, *p* = 0.9), as well as when the frequency of antibiotic use in the previous 72 h was compared (13.7 % *vs*. 24.4 %, *p* = 0.1).

**Table 1:** Demographic and clinical characteristics of children with non-severe, severe or very severe community-acquired pneumonia

Characteristics	Severity by the World Health Organization 2000 criteria		
	Non-severe (*n* = 53)	Severe (*n* = 111)	Very severe (*n* = 17)
Age (months)^a, b^	23 (13–35)	16 (9–26)	18 (13–26)
Length of disease (days)^a, c^	7 (5–13)	7 (4–11)	8 (4–11)
Tachypnoea^d^	53 (100.0)	97 (87.4)	15 (88.2)
Chest indrawing^d^	0	109 (98.2)	7 (41.2)
Supraclavicular recession^d^	0	4 (3.6)	0
Inability to drink^d^	0	0	17 (100.0)

^a^Median (interquartile range)

^b^Children with non-severe community-acquired pneumonia *versus* those with severe (*p* = 0.03) or *versus* those with severe and very severe CAP (*p* = 0.04)

^c^No significant difference was found

^d^Results in n (%)

Overall, 156 (86.2 %) patients had detected aetiology. Viral infection (*n* = 84; 46.4 %), bacterial infection (*n* = 26; 14.4 %) and mixed viral-bacterial infection (*n* = 46; 25.4 %) were identified. Table 2 depicts the overall frequency of the found aetiological agents along with the frequency of the positive laboratory tests. No significant difference was found in the frequency of detected aetiology when children who did or did not receive antibiotic in the 72 h prior to hospital admission were compared (86.8 % *vs*. 86.4 %, *p* = 0.9). Table 3 presents the comparison of different aetiologies among patients across distinct severity subgroups according to the WHO severity criteria in the 2000 decade. Aetiology was more likely to be determined in severe or very severe cases (89.8 %) compared to non-severe cases (77.4 %; *p* = 0.03). *C. trachomatis* infection was more frequent among severe (8.1 %) than non-severe (0 %) cases (*p* = 0.03). Rhinovirus was significantly uncommon (*p* = 0.03) among very severe cases (0 %) whereas 24.5 % of non-severe cases had rhinovirus found. Human metapneumovirus was detected only among severe (6.3 %) and very severe (5.9 %) patients. Pneumococcal infection increased across “non-severe” (13.2 %), “severe” (23.4 %), and “very severe” (35.3 %) cases (qui-squared test for trend *p* = 0.04). The frequency of bacterial and typical bacterial infection showed the same trend, being the statistical result borderline (*p* = 0.06). Infection by *C. pneumoniae* or *S. aureus* was not detected. Table 4 compares the frequency of the different aetiologies among patients across dinstict severity subgroups according to the WHO severity criteria published in the year 2013. In such analysis, rhinovirus was also significantly uncommon among severe cases in comparison with non-severe ones (0 % *vs*. 23.8 %; *p* = 0.03)

**Table 2:** Overall frequency of aetiological agents detected among 181 children hospitalized with community-acquired pneumonia

Aetiological agents	Frequency
*S. pneumoniae*	39 (21.5)
positive blood culture	8 (4.4)
positive blood PCR	7 (3.9)
invasive infection^a^	13 (7.2)
increased paired IgG titres only	26 (14.4)
*H. influenzae*	13 (7.2)
*M. pneumoniae*	11 (6.1)
*C. trachomatis*	9 (5.0)
*M. catarrhalis*	4 (2.2)
*S. negevensis* ^b^	3 (1.7)
Rhinovirus	39 (21.5)
RSV	36 (19.9)
antigen in NPA and increased paired IgG titres	17 (9.4)
antigen in NPA	11 (6.1)
increased paired IgG titres	8 (4.4)
Parainfluenza 1, 2, 3	35 (19.3)
antigen in NPA and increased paired IgG titres	11 (6.0)
antigen in NPA	2 (1.1)
increased paired IgG titres	22 (12.2)
Influenza A, B	15 (8.3)
antigen in NPA and increased paired IgG titres	3 (1.65)
antigen in NPA	3 (1.65)
increased paired IgG titres	9 (5.0)
Human bocavirus	17 (9.4)
low IgG avidity (<15)	17 (9.4)
positive IgM	16 (8.8)
qPCR in NPA >10E + 4/ml	15 (8.3)
positive qPCR in serum	4 (2.2)
IgG seroconversion	3 (1.65)
increased paired IgG titres	2 (1.1)
Adenovirus	10 (5.5)
antigen in NPA and increased paired IgG titres	3 (1.65)
antigen in NPA	1 (0.55)
increased paired IgG titres	6 (3.3)
Enterovirus	10 (5.5)
Human metapneumovirus	8 (4.4)

Results in n (%)

*RSV* respiratory syncytial virus

*NPA* nasopharyngeal aspirate

*qPCR* quantitative polymerase chain reaction

^a^Invasive disease was defined as positive blood culture (bacteraemia) and/or positive blood PCR for pneumococcus

^b^All three cases had IgM against *S. negevensis* detected

**Table 3:** Comparison between frequencies of different aetiologies among children with non-severe or severe and/or very severe community-acquired pneumonia according to the World Health Organization 2000 criteria

Aetiology	Overall frequency (*n* = 181)	Severity by the World Health Organization 2000 criteria						
		Non-severe (*n* = 53)	Severe or very severe (*n* = 128)	*p*	Severe (*n* = 111)	*p*	Very severe (*n* = 17)	*p*
Subgroups								
Detected	156 (86)	41 (77.4)	115 (89.8)	0.03	101 (91.0)	0.017	14 (82.4)	1
Viral infection	84 (46.4)	25 (47.2)	59 (46.1)	0.9	54 (48.6)	0.9	5 (29.4)	0.2
Bacterial infection	72 (39.8)	16 (30.2)	56 (43.8)	0.09	47 (42.3)	0.1	9 (52.9)	0.09
Typical bacterial infection	54 (29.8)	11 (20.8)	43 (33.6)	0.09	36 (32.4)	0.1	7 (41.2)	0.1
Atypical bacterial infection	18 (9.9)	5 (9.4)	13 (10.2)	0.9	11 (9.9)	0.9	2 (11.8)	1
Pathogens								
*S. pneumoniae*	39 (21.5)	7 (13.2)	32 (25.0)	0.08	26 (23.4)	0.1	6 (35.3)	0.07
Positive blood culture	8 (4.4)	3 (5.7)	5 (3.9)	0.7	3 (2.7)	0.4	2 (11.8)	0.6
Positive blood PCR	7 (3.9)	1 (1.9)	6 (4.7)	0.7	5 (4.5)	0.7	1 (5.9)	0.4
Invasive infection^a^	13 (7.2)	3 (5.7)	10 (7.8)	0.8	8 (7.2)	1	2 (11.8)	0.6
*H. influenzae*	13 (7.2)	4 (7.5)	9 (7.0)	1	8 (7.2)	1	1 (5.9)	1
*M. pneumoniae*	11 (6.1)	6 (11.3)	5 (3.9)	0.08	4 (3.6)	0.08	1 (5.9)	1
*C. trachomatis*	9 (5.0)	0	9 (7.0)	0.06	9 (8.1)	0.03	0	-
*M. catarrhalis*	4 (2.2)	0	4 (3.1)	0.3	4 (3.6)	0.3	0	-
*S. negevensis*	3 (1.7)	0	3 (2.3)	0.6	2 (1.8)	1	1 (5.9)	0.2
Rhinovirus	39 (21.5)	13 (24.5)	26 (20.3)	0.5	26 (23.4)	0.9	0	0.03
RSV	36 (19.9)	13 (24.5)	23 (18.0)	0.3	20 (18.0)	0.3	3 (17.6)	0.7
Parainfuenza 1, 2, 3	35 (19.3)	11 (20.8)	24 (18.8)	0.8	21 (18.9)	0.8	3 (17.6)	1
Influenza A, B	15 (8.3)	2 (3.8)	13 (10.2)	0.2	12 (10.8)	0.2	1 (5.9)	1
Human bocavirus	17 (9.4)	3 (5.7)	14 (10.9)	0.4	14 (12.6)	0.2	0	1
Adenovirus	10 (5.5)	2 (3.8)	8 (6.3)	0.7	7 (6.3)	0.7	1 (5.9)	1
Enterovirus	10 (5.5)	1 (1.9)	9 (7.0)	0.3	8 (7.2)	0.3	1 (5.9)	0.4
Human metapneumovirus	8 (4.4)	0	8 (6.3)	0.1	7 (6.3)	0.1	1 (5.9)	0.2

Results in n (%)

*RSV* respiratory syncytial virus

^a^Invasive disease was defined as positive blood culture (bacteraemia) and/or positive blood PCR for pneumococcus

**Table 4:** Comparison between frequencies of different aetiologies among children with non-severe or severe and/or very severe community-acquired pneumonia according to the World Health Organization 2013 criteria

Aetiology	Severity by the World Health Organization 2013 criteria		
	Non-severe (*n* = 164)	Severe (*n* = 17)	*p*
Subgroups			
Detected	142 (86.6)	14 (82.4)	0.7
Viral infection	79 (48.2)	5 (29.4)	0.1
Bacterial infection	63 (38.4)	9 (52.9)	0.2
Typical bacterial infection	47 (28.7)	7 (41.2)	0.3
Atypical bacterial infection	16 (9.8)	2 (11.8)	0.7
Pathogens			
*S. pneumoniae*	33 (20.1)	6 (35.3)	0.2
Positive blood culture	6 (3.7)	2 (11.8)	0.2
Positive blood PCR	6 (3.7)	1 (5.9)	0.5
Invasive infection^a^	11 (6.7)	2 (11.8)	0.4
*H. influenzae*	12 (7.3)	1 (5.9)	1.0
*M. pneumoniae*	10 (6.1)	1 (5.9)	1.0
*C. trachomatis*	9 (5.5)	-	1.0
*M. catarrhalis*	4 (2.4)	-	1.0
*S. negevensis*	2 (1.2)	1 (5.9)	0.3
Rhinovirus	39 (23.8)	-	0.03
RSV	33 (20.1)	3 (17.6)	1.0
Parainfuenza 1, 2, 3	32 (19.5)	3 (17.6)	1.0
Influenza A, B	14 (8.5)	1 (5.9)	1.0
Human bocavirus	17 (10.4)	-	0.4
Adenovirus	9 (5.5)	1 (5.9)	1.0
Enterovirus	9 (5.5)	1 (5.9)	1.0
Human metapneumovirus	7 (4.3)	1 (5.9)	0.6

Results in n (%)

*RSV* respiratory syncytial virus

^a^Invasive disease was defined as positive blood culture (bacteraemia) and/or positive blood PCR for pneumococcus

Because of the difference in age distribution in regard to different severity subgroups, we analysed age in regard to detected aetiology, pneumococcal, *C. trachomatis*, rhinovirus, or human metapneumovirus infection (Table 5). Age distribution was only different when children infected with human metapneumovirus were analysed: they were younger than those without it (*p* = 0.046).

**Table 5:** Comparison of age among children with or without specific aetiology

Characteristics	Compared subgroups		*p*
Detected aetiology	Yes (*n* = 156)	No (*n* = 25)	
Age (months)^a^	17 (10–27)	19 (11–30)	0.5
Pneumococcal infection	Yes (*n* = 39)	No (*n* = 142)	
Age (months)^a^	21 (11–31)	17 (10–27)	0.8
*C. trachomatis*	Yes (*n* = 9)	No (*n* = 172)	
Age (months)^a^	15 (4–23)	21 (10–28)	0.2
Rhinovirus infection	Yes (*n* = 39)	No (*n* = 142)	
Age (months)^a^	17 (9–34)	18 (11–27)	0.7
Human metapneumovirus infection	Yes (*n* = 8)	No (*n* = 173)	
Age (months)^a^	10 (5–18)	18 (11–28)	0.046

^a^Median (interquartile range)

Among children with detected aetiology, by grouping cases with sole bacterial infection together with cases with mixed viral-bacterial infection in a subgroup labeled bacterial infection, in multivariable analysis controlled for age, bacterial infection did not affect severity upon admission, using the WHO severity criteria availabe either in the 2000 decade or in 2013 (Table 6).

**Table 6:** Multivariable logistic regression analysis of bacterial infection and age (predictors) and severity (outcome) upon admission of hospitalized children with community-acquired pneumonia

Predictors	Severity by the World Health Organization 2000 criteria		unadjOR (95 % CI)	*p*	adjustOR (95 % CI)	*p*
	Non-severe (*n* = 53)	Severe or very severe (*n* = 128)				
Bacterial infection^a^	16 (30.2)	56 (43.8)	1.5 (0.7–3.1)	0.3	1.7 (0.8–3.5)	0.2
Age (months)^b^	23 (13–35)	16 (9–26)	0.8 (0.6–0.99)	0.04	0.7 (0.5–1.0)	0.07
	Severity by the World Health Organization 2013 criteria					
	Non-severe (*n* = 164)	Severe (*n* = 17)				
Bacterial infection^a^	63 (38.4)	9 (52.9)	2.3 (0.7–7.1)	0.2	2.3 (0.7–7.3)	0.2
Age (months)^b^	17 (10–27)	18 (13–24)	1.0 (0.6–1.6)	1.0	0.9 (0.6–1.6)	0.8

^a^Results in n (%)

^b^Median (interquartile range)

Out of 156 patients with established aetiology, 91 (58.3 %) had infection caused by one pathogen and 65 (41.7 %) had infection caused by two or more pathogens. In the former group, 25 (27.5 %) cases had bacterial infection and 66 (72.5 %) had viral infection. Conversely, in the latter group, 46 (70.8 %) had viral-bacterial infection, 18 (27.7 %) had viral-viral infection, and 1 (1.5 %) had bacterial-bacterial infection. Severe or very severe CAP was detected among 67 (73.6 %) patients with single infection, and among 48 (73.8 %) patients with co-infections (*p* = 1). Among patients with detected aetiology, after excluding cases with co-infection, the frequency of sole bacterial infection was different among children with non-severe (12.5 %), severe (29.3 %) or very severe (55.6 %) CAP cases (*p* = 0.04) (Table 7). Furthermore, by considering only the 91 cases with aetiology detected and without co-infection, sole bacterial infection was directly and independently associated with severity when the patients were grouped according to the WHO severity criteria available in the 2000 decade (Table 8). In this latter analysis, children with sole bacterial infection were compared with children with sole viral infection. The identified aetiological agents in the 25 cases with sole bacterial infection were *S. pneumoniae* (*n* = 12), *H. influenzae* (*n* = 4), *M. pneumoniae* (*n* = 4), *M. catarrhalis* (*n* = 2), *C. trachomatis* (*n* = 2), and *S. negevensis* (*n* = 1).

**Table 7:** Comparison between frequencies of sole bacterial and sole viral infection among 91 children hospitalized with community-acquired pneumonia and detected aetiology, after excluding cases with co-infection

Aetiology	Severity by the World Health Organization 2000 criteria		
	Non-severe (*n* = 24)	Severe (*n* = 58)	Very severe (*n* = 9)
Sole bacterial infection	3 (12.5)	17 (29.3)	5 (55.6)
Sole viral infection	21 (87.5)	41 (70.7)	4 (44.4)

Results in n (%)

*p* = 0.04 for the frequency of sole bacterial infection across three distinct severity categories among patients with sole aetiological agent infection

**Table 8:** Multivariable logistic regression analysis of bacterial infection and age (predictors) and severity (outcome) upon admission of hospitalized children with community-acquired pneumonia with documented infection by only one pathogen

Predictors	Severity by the World Health Organization 2000 criteria		unadjOR (95 % CI)	*p*	adjustOR (95 % CI)	*p*
	Non-severe (*n* = 24)	Severe or very severe (*n* = 67)				
Bacterial infection^a^	3 (12.5)	22 (32.8)	3.4 (0.9–12.7)	0.07	4.4 (1.1–17.6)	0.04
Age (months)^b^	19 (12–35)	17 (7–26)	0.8 (0.6–1.2)	0.3	0.7 (0.5–1.1)	0.1
	Severity by the World Health Organization 2013 criteria					
	Non-severe (*n* = 82)	Severe (*n* = 9)				
Bacterial infection^a^	20 (24.4)	5 (55.6)	3.9 (0.9–15.8)	0.06	3.8 (0.9–16.0)	0.07
Age (months)^b^	18 (10–26)	19 (14–31)	1.2 (0.7–2.1)	0.5	1.1 (0.6–1.9)	0.9

^a^Results in n (%)

^b^Median (interquartile range)

Cases with incomplete specimen collection (Fig. 1) were at random. In order to address systematic bias, the whole analysis was repeated with the inclusion of these cases and the results were the same (Additional file 1).

## Discussion

This study showed that respiratory viral infections were detected in a reasonable proportion of cases in each severity subgroup. The frequencies of these infections did not differ across the distinct severity categories. Notably, one third to half of the cases classified as severe CAP had respiratory viral infections (Tables 3 and 4). Human metapneumovirus was the only viral pathogen exclusively found in the severe or very severe categories (Table 3). However, the difference was not significant. Pneumococcal infection increased across “non-severe”, “severe”, and “very severe” cases (Table 3). Only after excluding co-infections, sole bacterial infection was significantly more frequent among children with severe or very severe CAP (Table 8). No difference was found in the frequency of invasive typical bacterial infection (Tables 3 and 4).

Viral CAP has been recognized as a frequent entity among children, with an estimated occurrence of 100 million cases annually of which one third presents viral-bacterial co-infection [20]. Molecular diagnostics have played a fundamental role in obtaining these findings [21]. Indeed, viruses currently account for the largest proportion of CAP in preschool children in both developed and developing countries [22]. However, the pathogenesis and clinical impact of viral lung infection is not well understood [23]. Interestingly, herein, human metapneumovirus infection was only identified among severe or very severe cases. Human metapneumovirus has been identified as a single pathogen in childhood CAP [24] and it was found more frequently among children with alveolar CAP than in controls [25]. Disease caused by human metapneumovirus may be severe enough to require hospitalization or even admission to a paediatric intensive care unit [26]. Our results support the importance of viral respiratory pathogens contributing to CAP severity.

Noteworthily, pneumococcal infection frequency increased across “non-severe”, “severe”, and “very severe” cases. Additionally, after excluding the cases with co-infection, sole bacterial infection was independently associated with severity (Table 8), that is, sole bacterial infection is associated with severe CAP when compared with sole viral infection. This finding suggests that bacteria may be more aggressive than viruses when there is one sole pathogen related to the CAP illness. These results are in accordance with the classical association between clinical severity and bacterial infection [4]. A recent systematic and meta-analysis reported an estimated increase in the rate of bacterial infection among severe cases in relation to patients with non-severe CAP. This rate was found to be even more important in the subgroup of the children who eventually die of the disease [27]. Nonetheless, no differences were found in the frequency of invasive bacterial disease (Tables 3 and 4). Figures from studies conducted among adults have shown that pneumococcal bacteraemia increases the risk of mortality and extra-pulmonary involvement in patients with pneumococcal CAP which was attributable to their older age and higher frequency of underlying medical conditions [28]. Host factors related to severity of disease have been recognized as confounders of the association between pneumococcal bacteraemia (invasive disease) and poor outcomes [29]. Herein, we excluded children with possible confounders for opportunistic infections and with co-morbidities.

Aetiology was more likely to be determined in severe or very severe cases (Table 3). Despite using an expanded range of diagnostic tests to search for each pathogen by different tools, aetiology was not detected in all cases. This finding may be attributable to unrecognized aetiological agents, or limitation of the employed methods, or both. Patients diagnosed as either severe or very severe cases were younger than those non-severe patients (Table 1). During the first three years of life whilst the immune system is maturing, particularly the production of IgG, the host is more vulnerable to infections [30]. Moreover, the first two years are often regarded as the most vulnerable period, especially for infections due to capsulated bacteria as pneumococcus and capsulated *H. influenzae*.

Our results must be viewed with caution. A higher number of cases would provide more precise results for comparing each pathogen’s frequencies. That is, low statistical power due to relatively small sample size is the main limitation of this study. Although a wide range of tests to investigate a big number of aetiological agents was performed, it was difficult to assign causality even if aetiology was known, especially when there were co-detections. Aetiology was established as probable because lung tissue was not studied, for ethical reasons. However, different techniques were used, sometimes searching for the same pathogen to strengthen each result. Besides searching for the pathogen (by blood culture or PCR or antigen detection) we also searched for the host’s response to the presence of the pathogen. Pneumococcal aetiology was assessed by blood culture, paired pneumococcal antibodies titres and buffy-coat PCR. This approach is better than culturing or performing PCR of respiratory samples as *S. pneumoniae* commonly colonizes children’s nasopharynx [31]. Antibody increase in paired serum samples due to acquisition of a new pneumococcal colonizing strain has been described to occur in <1 % to 3 % of the healthy children [32]. However, the final roles of pneumococcal serology and buffy-coat PCR are still open for discussion. The roles of non-capsulated *H. influenzae*, *M. catarrhalis*, and *S. negevensis* are less clear in the aetiology of childhood CAP. Likewise, serological diagnoses of Mycoplasma and Chlamydia infections are difficult. This approach may be acceptable, as no better approaches are currently available, particularly because Mycoplasma strains found in the upper respiratory samples can be detected by PCR without any relation to the current illness [33]. Additionally, the lack of pneumococcal and influenza vaccines resulted in difficulty to extend our findings to regions with high vaccine coverage. As such, in these regions, it is expected that the role of respiratory viruses is even more remarkable among children hospitalized with CAP, whilst pneumococcal infection is supposedly less frequent. In a recent study on CAP among 2,222 U.S. hospitalized children, viral (66 %), bacterial (8 %), or viral-bacterial (7 %) infections were reported [34]. However, the relative positivity rates between non-pneumococcal and non-influenza pathogens reported herein are still of interest. Additionally, it is important to note that severity was assessed on the basis of clinical findings present upon admission and not inferred from the evolution of the patients, as well as, severity was measured by using standardized protocols by a blinded investigator.

The WHO changed the severity CAP criteria in 2013 in order to make feasible the treatment at home of cases previously labeled as severe [19]. Several differences in the aetiologies frequencies were found when we used the WHO severity criteria available in the 2000 decade [18], but not all these differences were found when we used the WHO severity criteria published in 2013 [19] (Tables 3, 4 and 8). This finding raises the question about possible true differences among the subgroups formed by using the 2000 WHO severity criteria, as WHO changed these criteria to facilitate treatment logistic.

## Conclusions

Respiratory viral infections were detected in a reasonable proportion of cases in each severity subgroup. A high proportion of patients with viral detections supports the need for more antiviral medicines and vaccines. Bacterial infection, particularly pneumococcal infection, was more likely among severe/very severe cases. This finding supports the use of empiric antibiotics to treat these patients, as it has been done in clinical practice.

## Abbreviations

95 % CI, 95 % confidence interval; CAP, community-acquired pneumonia; ELISA, enzyme immune assay; IQR, interquartile range; MIF, microimmunofluorescence; NPA, nasopharyngeal aspirate; PCR, polymerase chain reaction; RSV, respiratory syncytial virus; WHO, World Health Organization.

## Additional file

Additional file 1: Dataset of children hospitalized with community-acquired pneumonia in distinct severity categories with aetiology results. Demographic, clinical, and aetiology data on children hospitalized with community-acquired pneumonia. (XLS 219 kb)

## References

[1] Walker CL, Rudan I, Liu L, Nair H, Theodoratou E, Bhutta ZA (2013). Global burden of childhood pneumonia and diarrhoea. Lancet.

[2] Shann F (1986). Etiology of severe pneumonia in children in developing countries. Pediatr Infect Dis.

[3] Vuori-Holopainen E, Peltola H (2001). Reappraisal of lung tap: review of an old method for better etiologic diagnosis of childhood pneumonia. Clin Infect Dis.

[4] Gilani Z, Kwong YD, Levine OS, Deloria-Knoll M, Scott JA, O’Brien KL (2012). A literature review and survey of childhood pneumonia etiology studies: 2000-2010. Clin Infect Dis.

[5] Johansson N, Kalin M, Tiveljung-Lindell A, Giske CG, Hedlund J (2010). Etiology of community-acquired pneumonia: increased microbiological yield with new diagnostic methods. Clin Infect Dis.

[6] Howie SR, Morris GA, Tokarz R, Ebruke BE, Machuka EM, Ideh RC (2014). Etiology of severe childhood pneumonia in The Gambia West Africa determined by conventional and molecular microbiological analyses of lung and pleural aspirate samples. Clin Infect Dis.

[7] Nohynek H, Eskola J, Kleemola M, Jalonen E, Saikku P, Leinonen M (1995). Bacterial antibody assays in the diagnosis of acute lower respiratory tract infection in children. Pediatr Infect Dis J.

[8] Saukkoriipi A, Palmu A, Kilpi T, Leinonen M (2002). Real-time quantitative PCR for the detection of *Streptococcus pneumoniae* in the middle ear fluid of children with acute otitis media. Mol Cell Probes.

[9] Atkinson TP, Waites KB (2014). Mycoplasma pneumoniae infections in childhood. Pediatr Infect Dis J.

[10] Morré SA, Munk C, Persson K, Krüger-Kjaer S, van Dijk R, Meijer CJ (2002). Comparison of three commercially available peptide-based immunoglobulin G (IgG) and IgA assays to microimmunofluorescence assay for detection of Chlamydia trachomatis antibodies. J Clin Microbiol.

[11] Yamaguchi T, Yamazaki T, Inoue M, Mashida C, Kawagoe K, Ogawa M (2005). Prevalence of antibodies against Simkania negevensis in a healthy Japanese population determined by the microimmunofluorescence test. FEMS Immunol Med Microbiol.

[12] Mäkelä MJ, Puhakka T, Ruuskanen O, Leinonen M, Saikku P, Kimpimäki M (1998). Viruses and bacteria in the etiology of the common cold. J Clin Microbiol.

[13] Korppi M, Jartti T, Hedman K, Söderlund-Venermo M (2010). Serologic diagnosis of human bocavirus infection in children. Pediatr Infect Dis J.

[14] Nascimento-Carvalho CM, Ribeiro CT, Cardoso MR, Barral A, Araújo-Neto CA, Oliveira JR (2008). The role of respiratory viral infections among children hospitalized for community-acquired pneumonia in a developing country. Pediatr Infect Dis J.

[15] Nascimento-Carvalho CM, Cardoso MR, Paldanius M, Barral A, Araújo-Neto CA, Saukkoriipi A (2009). Simkania negevensis infection among Brazilian children hospitalized with community-acquired pneumonia. J Infect.

[16] Nascimento-Carvalho CM, Cardoso MR, Ruuskanen O, Lappalainen M (2011). Sole infection by human metapneumovirus among children with radiographically diagnosed community-acquired pneumonia in a tropical region. Influenza Other Respir Viruses.

[17] Nascimento-Carvalho CM, Cardoso MR, Meriluoto M, Kemppainen K, Kantola K, Ruuskanen O (2012). Human bocavirus infection diagnosed serologically among children admitted to hospital with community-acquired pneumonia in a tropical region. J Med Virol.

[18] World Health Organization (2000). Management of the child with a serious infection or severe malnutrition: guidelines for care at the first-referral level in developing countries.

[19] World Health Organization (2013). Pocket book of hospital care for children. Guidelines for the management of common childhood illnesses.

[20] Ruuskanen O, Lahti E, Jennings LC, Murdoch DR (2011). Viral pneumonia. Lancet.

[21] Nolte FS (2008). Molecular diagnostics for detection of bacterial and viral pathogens in community-acquired pneumonia. Clin Infect Dis.

[22] Mameli C, Zuccotti GV (2013). The impact of viral infections in children with community-acquired pneumonia. Curr Infect Dis Rep.

[23] Ruuskanen O, Jarvinen A (2014). What is the real role of respiratory viruses in severe community-acquired pneumonia?. Clin Infec Dis.

[24] Woods CR, Bryant KA (2013). Viral infections in children with community-acquired pneumonia. Curr Infect Dis Rep.

[25] Wolf DG, Greenberg D, Shemer-Avni Y, Givon-Lavi N, Bar-Ziv J, Dagan R (2010). Association of human metapneumovirus with radiologically diagnosed community-acquired alveolar pneumonia in young children. J Pediatr.

[26] Principi N, Esposito S (2014). Paediatric human metapneumovirus infection: epidemiology, prevention and therapy. J Clin Virol.

[27] Rudan I, O’Brien KL, Nair H, Liu L, Theodoratou E, Qazi S (2013). Epidemiology and etiology of childhood pneumonia in 2010: estimates of incidence, severe morbidity, mortality, underlying risk factors and causative pathogens for 192 countries. J Glob Health.

[28] Lin SH, Lai CC, Tan CK, Liao WH, Hsueh PR (2011). Outcomes of hospitalized patients with bacteraemic and non-bacteraemic community-acquired pneumonia caused by Streptococcus pneumoniae. Epidemiol Infect.

[29] Bordón J, Peyrani P, Brock GN, Blasi F, Rello J, File T (2008). The presence of pneumococcal bacteremia does not influence clinical outcomes in patients with community-acquired pneumonia: results from the Community-Acquired-Pneumonia Organization (CAPO) International Cohort study. Chest.

[30] Ishimine P (2006). Fever without source in children 0 to 36 months of age. Pediatr Clin North Am.

[31] Bogaert D, Sluijter M, Toom NL, Mitchell TJ, Goessens WH, Clarke SC (2006). Dynamics of pneumococcal colonization in healthy Dutch children. Microbiology.

[32] Korppi M, Leinonen M, Ruuskanen O (2008). Pneumococcal serology in children’s respiratory infections. Eur J Clin Microbiol Infect Dis.

[33] Spuesens EB, Fraaij PL, Visser EG, Hoogenboezem T, Hop WC, van Adrichem LN (2013). Carriage of Mycoplasma pneumoniae in the upper respiratory tract of symptomatic and asymptomatic children: an observational study. PLoS Med.

[34] Jain S, Williams DJ, Arnold SR, Ampofo K, Bramley AM, Reed C (2015). Community-acquired pneumonia requiring hospitalization among U.S. children. N Engl J Med.

